# IL-32 promoter SNP rs4786370 predisposes to modified lipoprotein profiles in patients with rheumatoid arthritis

**DOI:** 10.1038/srep41629

**Published:** 2017-01-30

**Authors:** Michelle S. M. A. Damen, Rabia Agca, Suzanne Holewijn, Jacqueline de Graaf, Jéssica C. Dos Santos, Piet L. van Riel, Jaap Fransen, Marieke J. H. Coenen, Mike T. Nurmohamed, Mihai G. Netea, Charles A. Dinarello, Leo A. B. Joosten, Bas Heinhuis, Calin D. Popa

**Affiliations:** 1 Department of Internal Medicine and Radboud Center for Infectious Diseases (RCI), Radboud University Medical Center, 6525 GA Nijmegen, The Netherlands; 2 Amsterdam Rheumatology immunology Center, Department of Rheumatology, location CU University Medical Center and Reade, Amsterdam, The Netherlands; 3 Rijnstate Ziekenhuis, Arnhem, The Netherlands; 4 Instituto de Patologia Tropical e Saúde Pública, Universidade Federal de Goiás, Brazil; 5 Department of Rheumatology, Bernhoven Ziekenhuis, Uden, The Netherlands; 6 Department of Rheumatology, Radboud University Medical Center, 6525GA Nijmegen, The Netherlands; 7 Radboud Institute for Health Sciences, Department of Human Genetics, Radboud university medical center, Nijmegen, The Netherlands; 8 School of Medicine, Division of infectious diseases, University of Colorado Denver, Aurora, Colorado 80045, United States of America

## Abstract

Patients with rheumatoid arthritis (RA) are at higher risk of developing cardiovascular diseases (CVD). Interleukin (IL)-32 has previously been shown to be involved in the pathogenesis of RA and might be linked to the development of atherosclerosis. However, the exact mechanism linking IL-32 to CVD still needs to be elucidated. The influence of a functional genetic variant of IL-32 on lipid profiles and CVD risk was therefore studied in whole blood from individuals from the NBS cohort and RA patients from 2 independent cohorts. Lipid profiles were matched to the specific IL-32 genotypes. Allelic distribution was similar in all three groups. Interestingly, significantly higher levels of high density lipoprotein cholesterol (HDLc) were observed in individuals from the NBS cohort and RA patients from the Nijmegen cohort homozygous for the C allele (p = 0.0141 and p = 0.0314 respectively). In contrast, the CC-genotype was associated with elevated low density lipoprotein cholesterol (LDLc) and total cholesterol (TC) in individuals at higher risk for CVD (plaque positive) (p = 0.0396; p = 0.0363 respectively). Our study shows a functional effect of a promoter single-nucleotide polymorphism (SNP) in *IL32* on lipid profiles in RA patients and individuals, suggesting a possible protective role of this SNP against CVD.

In patients with rheumatoid arthritis (RA), cardiovascular disease (CVD) represents the leading cause of death^1^,^2^. Various studies have demonstrated that besides behavioral risk factors and dyslipidemia, inflammation also plays a crucial role in the increased risk for CVD^3^. Additionally, inflammatory pathways in RA but also other chronic inflammatory diseases, including psoriasis, have been proposed to accelerate atherogenesis contributing to the increased CVD risk^4^,^5^,^6^. These patients are continuously exposed to varying levels of inflammatory mediators (e.g. cytokines) that may alter traditional CVD risk factors, including the lipid pattern, both at the concentration and composition level^7^,^8^. Normally, a pro-atherogenic lipid profile consists of an increased total cholesterol (TC), low-density lipoprotein cholesterol (LDLc), triglycerides (TG), and a decreased high-density lipoprotein cholesterol (HDLc). However, in RA patients the lipid profile varies throughout different stages of the disease^9^,^10^. Particularly during active disease, these patients have low TC and LDLc levels, while their CVD risk is increased. Hence, due to the changeability of inflammatory activity and anti-inflammatory medication, the individual lipid profiles may frequently fluctuate during the course of disease making it hard to draw conclusions about the impact of these changes on CVD risk^11^. Of all lipids, HDLc is the most susceptible to inflammatory changes in terms of both concentration as well as composition^12^,^13^,^14^. In line with this, it was previously shown that HDLc becomes less anti-atherogenic or even pro-atherogenic in RA patients with an increased inflammatory status^11^.

Recently, IL-32 has been demonstrated to be an important key modulator of inflammation in RA^15^. In a previous study from our group, IL-32 was found to be highly expressed in synovial tissues from patients with moderate and severe rheumatoid arthritis and it was strongly correlated with the severity of joint inflammation. IL-32 can be induced by TNF and can on its own further potentiate TNF expression^16^,^17^. Given this fact and the well-known roles of TNF in both RA and atherosclerosis, IL-32 was recently proposed to contribute to the development of atherosclerotic plaques^18^,^19^,^20^. In 2009 Dinarello *et al*. described IL-32 as a critical regulator of endothelial cell function, possibly promoting atherosclerosis by potentiating IL-1β-induced ICAM-1 and by producing pro-inflammatory cytokines in these cells^21^. This pro-atherogenic role of IL-32 was further supported by a recent report, which showed enhanced IL-32 expression in atherosclerotic arterial vessel walls^22^. Additionally, IL-32 was found to be expressed by macrophages, with highly increased expression after stimulation of these cells with pro-inflammatory components that were previously appointed to be involved in atherosclerosis (e.g. toll-like receptor (TLR) 3 agonist Poly I:C and interferon-gamma (IFNγ))^23^,^24^,^25^. Moreover, macrophages are known to play an important role in controlling cholesterol levels in blood vessel walls as they engulf cholesterol, become foam cells, and take part in reverse-cholesterol transport (RCT)^26^,^27^. Knowing that IL-32 expression can be highly induced in these cells upon inflammation, one can argue a role for IL-32 in cholesterol metabolism. Despite the suggested role of IL-32 in inflammation, CVD and disease progression in RA, studies investigating the IL-32 protein function and IL-32 gene polymorphisms with respect to these outcomes in RA are scarce^22^. However, our group recently showed that a single-nucleotide polymorphism (SNP) in the promoter region of the IL-32 gene seemed to be associated with lower basal expression of IL-32β in peripheral blood mononuclear cells (PBMCs) of RA patients^28^. The same data showed lower pro-inflammatory cytokine production in PBMCs after stimulation with various compounds in patients bearing the CC genotype^28^. Additionally, various studies, including genome-wide association studies (GWAS) analyses, show effects of gene polymorphisms in for example TNF, CD247 and anti-cyclic citrullinated peptide (anti-CCP) RA and CVD^18^,^29^,^30^,^31^. The present study therefore aims to investigate the functional implications of a SNP in the IL-32 gene on the lipid profile and CVD risk in RA patients.

## Results

### Genotype distribution of an *IL32* promoter SNP is comparable between individuals from the NN cohort and RA patients

**Figure 1:**
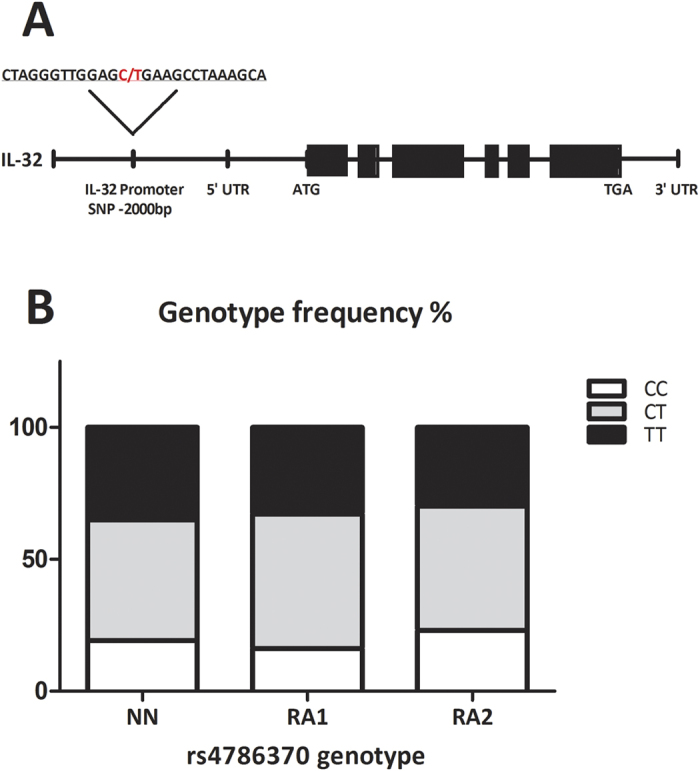
The IL-32 promoter SNP. (**A**) Location of the IL32 promoter SNP within the IL32 region on chromosome 16. (**B**) Genotype frequencies of the *IL32* rs4786370 promoter SNP in individuals from the NN cohort (NN; CC:19.2%, CT:45.7%, TT:35%), RA patients from the Radboudumc Nijmegen (RA1; CC:16.1%, CT:51%, TT:32.9%) and RA patients from the Reade clinic Amsterdam (RA2; CC:23%, CT:47.1%, TT:29.9%). Total number of patients per cohort; NN:#234, RA1:#292, RA2:#348. Chi-square analysis (IBM SPSS Statistics v.22) showed no significant differences in genotype distribution between the cohorts.

The genotype distribution of the *IL32* promoter SNP (rs4786370) (Fig. 1A) was compared between three different cohorts. One cohort consisted of individuals from the NBS NIMA cohort (NN). The other two cohorts consisted of RA patients, one group of patients treated at the Radboudumc Nijmegen (RA1) and the other at the Reade Clinic in Amsterdam (RA2). No significant differences in genotype frequencies were observed between the three cohorts (Fig. 1B).

### The *IL32* promoter SNP affects HDLc levels in both individuals from the NN cohort and RA patients

**Figure 2:**
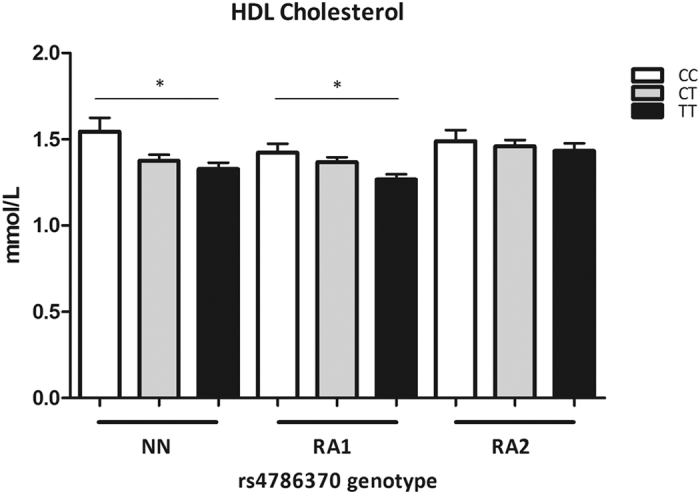
HDL cholesterol levels. A HDL cholesterol levels stratified for the *IL-32* promoter SNP (rs4786370) genotype in individuals from the NN cohort (TT#82, CT:#107, CC:#45, NN cohort) and RA patients from two different cohorts (RA1; TT:#96, CT:#149, CC:#47 and RA2; TT:#104, CT:#164, CC:#80). A significant induction in HDL cholesterol was observed in both individuals from the NN cohort and RA patients of the RA1 cohort carrying the C allele (NN; p = 0.0141, RA1; p = 0.0314 and RA2; p = 0.8450). Values are expressed as means ± SEM. *p*-values are calculated using Mann-Whitney *U*-test, *p < 0.05 Graphpad prism v5.03.

We studied the concentration of HDLc in each cohort because of its importance in development of cardiovascular disease. As shown in Fig. 2, in all three cohorts individuals having the CC genotype show higher levels of HDLc compared to either individuals with the CT and TT genotype with a significant increase in HDLc within the NN and RA1 cohort (p = 0.0141 and p = 0.0314 respectively). Further analysis was performed focussing on gender differences within each cohort (NN: Female:#120; Male:#114 RA1: Female:#155; Male:#137 and RA2: Female:#232; Male:#121). Only within the RA2 cohort there was a larger difference in number of females to males, nevertheless in none of the cohorts gender affected the HDLc (data not shown). Moreover, individuals carrying one C-allele show a small increase in HDLc levels. Concluding, the C-allele for the *IL-32* promoter SNP is linked to an increase in HDLc levels independent of gender or having RA.

### HDLc levels are affected by the *IL32* promoter SNP independent of the presence of plaques

**Figure 3:**
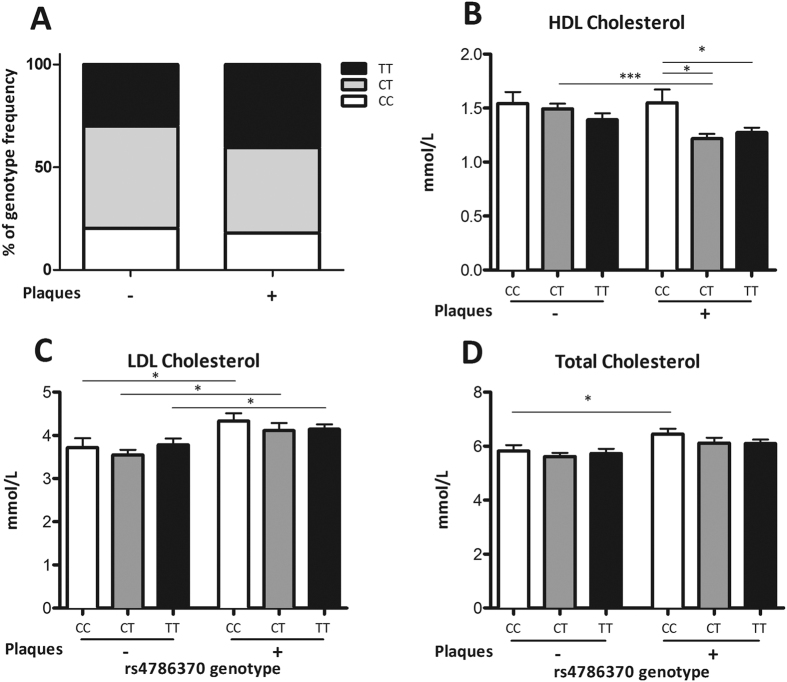
Lipoproteins and presence of plaques. (**A**–**D**) Lipoproteins stratified for *IL-32* promoter SNP rs4786370 and the presence or absence of plaques in individuals from the NN cohort. Genotype frequency, HDL cholesterol, LDL cholesterol and total cholesterol concentrations were determined in these individuals. Number of individuals CC−:25, CC+:20, CT−:61, CT+:46, TT−:37, TT+:45. Chi-square analysis (IBM SPSS Statistics v.22) showed no significant differences in genotype distribution between the groups. Significant differences were observed in HDLc (CT vs CTplaques p = 0.0004; CCplaques vs CTplaques p = 0.0069; CCplaques vs TTplaques p = 0.0256), LDLc (CC vs CCplaques p = 0.0346; CT vs CTplaques p = 0.0116; TT vs TTplaques p = 0.0396) and TC (CC vs CCplaques p = 0.0363). Values are expressed as means ± SEM. *p*-values are calculated using Mann-Whitney *U*-test, *p < 0.05 Graphpad prism v5.03.

Within the NN-cohort we were able to stratify the genotype frequency and cholesterol levels for the presence or absence of plaques detected by ultrasound (Fig. 3A–D). The percentage of individuals with the CC genotype seemed to be higher in the plaque negative group compared to the plaque positive group; 20% vs. 18% respectively although this did not reach statistical significance (Fig. 3A). Additionally, individuals carrying the CC or CT genotype showed higher HDLc levels than individuals with the TT genotype independent of having plaques. Nevertheless, individuals with the CT genotype and having a plaque showed a decrease in HDLc (CT− versus CT+: p < 0.0004) (Fig. 3B).

Both LDLc and TC were not affected by the SNP in *IL32* itself. However, higher levels of both LDLc and TC were observed in individuals who were found positive for plaques (Fig. 3C,D).

### HDLc is linked to the *IL32* promoter SNP and the prevalence of CVD events in RA patients

**Figure 4:**
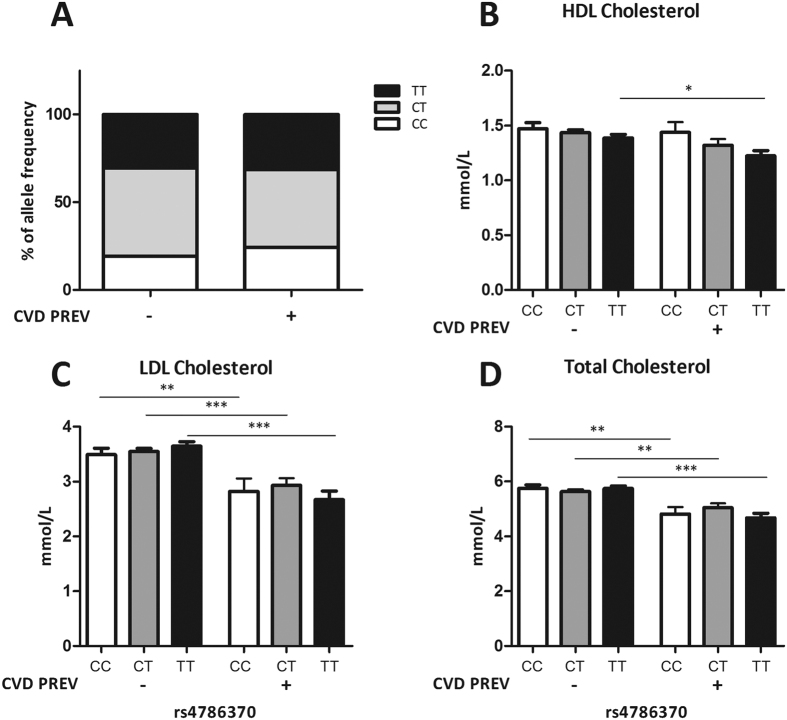
Lipoproteins and CVD events. (**A–D**) Lipoproteins stratified for IL-32 promoter SNP rs4786370 and the presence or absence of an previously described CVD. Allele frequency, HDL Cholesterol, LDL Cholesterol and Total Cholesterol concentrations were determined in patients with RA from the RA1 (Radboud University Medical Center Nijmegen) and RA2 cohort (Reade center Amsterdam). Number of individuals: CC−:98(19.1%), CC+:27(24.3%), CT−:259(50.4%), CT+:49(44.1%), TT−:157(30.5%), TT+:35(31.5%). Chi-square analysis (IBM SPSS Statistics v.22) showed no significant differences in allelic distribution between the cohorts. There was a significant difference in HDLc between the individuals with the TT genotype versus TT genotype + previous CVD; p = 0.0293. Values are expressed as means ± SEM. *p*-values are calculated using Mann-Whitney *U*-test, *p < 0.05 Graphpad prism v5.03.

CVD is a common problem in RA and the composition of cholesterol levels plays a crucial role herein. To determine if the *IL-32* promoter SNP was involved in both these parameters, genotypes and cholesterol levels were determined in RA patients (RA1 and RA2 cohort) with versus without a history in CVD (Fig. 4A–D). No differences were observed in genotype distribution between the two groups (Fig. 4A). Even so, HDLc levels were lower in individuals with a history of CVD, reaching lowest concentrations in individuals with the TT genotype (TT− versus TT+ p = 0.0293) (Fig. 4B). In contrast, patients carrying the CC genotype showed the highest levels of HDLc as was also observed within the individuals from the NN cohort (Fig. 3B). Besides, lower levels of LDLc and TC were observed in individuals with a history of CVD (Fig. 4C,D). This was completely opposite from what was found in individuals from the NN cohort as shown in Fig. 3C,D. Overall, patients with a history of CVD and carrying the TT genotype had lowest HDLc, LDLc and TC (Fig. 4B–D).

## Discussion

The major novel findings of this study are that a promoter SNP in the IL32 gene is equally distributed in individuals from the NN cohort as in RA patients, but at the same time that this SNP causes an increase in HDLc in both groups. This effect doesn’t seem to be influenced by disease activity, the use of medication, the presence of plaques or a history of CVD (data not shown). In contrast, other lipid concentrations like LDLc and TC are not affected by the SNP but are linked with plaques or a history of CVD. This might further expand the knowledge about the underlying mechanisms for CVD in RA patients^32^,^33^,^34^.

Although chronic inflammation is known to affect cholesterol concentration in the body resulting in lower HDLc and LDLc levels, some studies have not been able to show the benefit of increasing HDLc^34^,^35^,^36^,^37^,^38^. Nevertheless, HDLc does play a role in lowering the risk for CVD, especially in individuals with chronic inflammatory diseases like RA^39^,^40^. In RA there seems to be a tight relationship between the levels of HDLc, LDLc and disease activity, with lower levels during periods of active disease^41^,^42^,^43^. Even though this might speak against an increased risk for CVD, it is more the ratio between the levels of these different lipids (and probably their composition/function) that matters when assessing CVD risk. The fact that HDLc is decreased to a greater extent than the TC in these patients, results in a higher atherogenic index (the ratio between TC and HDLc) and therefore an increased CVD risk^37^,^44^. In addition, HDLc is less capable of exercising its anti-atherogenic functions if inflammation levels are still uncontrolled^8^. Conversely, when RA patients receive standard treatment including DMARDs and biological agents like anti-TNF therapy, cholesterol levels might increase, which correlates with the level of suppression of their disease activity^44^,^45^. In our study, no differences were observed in disease activity between genotypes concluding that the variation in HDLc was not caused by difference in disease activity (Supplementary Fig. S1A,B). Nevertheless, CVD is still the number one cause of death in RA, showing the importance of exploring how cholesterol metabolism and regulation is affected in these patients.

Genetic factors can also play a role in the development of the accelerated atherogenesis observed in RA patients as was described previously. Some studies describe that several gene polymorphisms for example in TNFα and NFkB1 have been found associated with an increased risk of CVD or subclinical atherosclerosis in RA^18^,^46^,^47^. Moreover, GWAS studies show many different gene polymorphisms related to RA, also finding new polymorphisms which were not yet described in other studies like polymorphisms in IL6ST, IRF5 and PTPN2^29^,^48^. In at least one study which performed GWAS analyses, IL-32 was present in the data which could suggest a role for IL-32 in the disease^49^. In contrast, other studies investigating gene polymorphisms in IL-6 for example, were not able to link these to an increased risk of subclinical atherosclerosis or CVD in RA when it was hypothesized that these could be associated^50^,^51^.

IL-32 has previously been described to play a role in the pathogenesis of RA and additionally it has been suggested that IL-32 plays a role in atherosclerosis, presumably contributing to the increased CVD risk in this population. As mentioned earlier, recent data showed that a SNP in the promoter region of the *IL-32* gene seemed to be associated with lower basal expression of IL-32β and lower pro-inflammatory cytokine production in PBMCs of RA patients bearing the CC-genotype^28^. Therefore, we hypothesized that there is also a genetic polymorphism in the promoter region of IL-32 that could affect and possibly lower the CVD risk in RA patients. To our knowledge, this is the first study demonstrating that a functional SNP in *IL-32* is linked to an increase in HDLc in RA patients and individuals from the NN cohort, suggesting IL-32 by itself can affect cholesterol metabolism. Individuals carrying the CT genotype already showed higher HDLc levels compared to individuals bearing the TT genotype, while having the CC genotype results in even higher HDLc. This suggests that having one C-allele might already affect IL-32 expression in a way that results in higher HDL cholesterol. The increased HDLc concentrations in individuals bearing the C-allele might be due to lower levels of TNFα, which is known to suppress cholesterol synthesis^52^. Another possible explanation could be that expression of a specific IL-32 isoform affects intracellular pathways resulting in for example higher cholesterol efflux or increased level of apoA-I (the main protein component of HDLc). The SNP however only seems to affect HDLc since no observation was made in individuals from the NN cohort or RA patients linking the SNP to lower or higher LDLc or TC. In our study, RA patients with a history of CVD had lower LDLc and TC compared to patients without a history of CVD and also lower levels than individuals from the NN cohort. This might be explained by the fact that these RA patients probably received statins after going through an event.

Our study has several limitations. Most of them are related to the difficulties of comparing the three heterogeneous cohorts. Various measurements like blood pressure or underlying disease (like diabetes) were not notified for all patients or individuals within the cohorts making it impossible to correct for all these factors.

When taken together, we suggest a novel role for a genetic polymorphism in the promoter region of IL-32 causes an increase in HDLc levels in individuals from the NN cohort and RA patients. This might have functional effects leading to a lower prevalence of carotid artery plaques. This has not been investigated in the present study, but should be taken into account in future investigations. Our results lead to the assumption that IL-32 is a previously unrecognized cytokine involved in the process of inflammation and cholesterol metabolism. However, currently we are still investigating the exact mechanism behind the role of IL-32 in cholesterol metabolism regulation.

## Methods

### Patient cohorts

From the total number of participants from the NBS NIMA study, only a subgroup of 234 participants, from whom the IL32 promoter SNP was determined, were included in this study. The NBS is a population-based survey as described before^53^. Participants aged 50–70 yr were asked to visit the hospital to perform six non-invasive measurements of atherosclerosis (NIMA) including pulse wave velocity (PWV), augmentation index (Aix), intima-media thickness (IMT), plaque thickness, ankle-brachial index (ABI) at rest and after exercise. Additionally, fasting venous blood samples were collected. All participants filled out a questionnaire about their previous history of vascular disease, medication use, smoking habits, and exercise habits. Prevalent CVD was defined as a reported myocardial infarction (MI), transient ischemic attack (TIA), stroke (CVA), peripheral arterial disease (PAD), coronary artery bypass or angioplasty, or treated angina. The Medical Ethics Committee of the Radboud university medical center, Nijmegen (nr. 2010-397), The Netherlands approved the study protocol, and all participants provided written informed consent^54^. Experiments were conducted according to the principles expressed in the Declaration of Helsinki.

Patients with RA who fulfilled the 1987 ACR criteria and/or the 2010 ACR/EULAR classification criteria for RA were recruited from the Radboudumc in Nijmegen, The Netherlands. These patients underwent a screening program of their CVD risk factors between July 2011 and August 2012. Disease-related parameters, lipid profile and history of cardiovascular events were registered (Table 1). In addition, the Nijmegen inception cohort database was checked for patients who had already been screened previously. The Nijmegen inception cohort is a prospective study that started in 1985 which includes regular visits for disease related parameters and blood samples in patients with RA. Eventually, 297 patients have been identified as participants of both the inception cohort and the CVD screening program. These patients were included in this study. The stored blood samples of the inception cohort were used for the determination of the SNP in the *IL-32* gene.

The CARdiovascular research and RhEumatoid arthritis (CARRÉ) study is an ongoing prospective cohort investigating cardiovascular (CV) disease and CV risk factors in 353 patients with RA (CCMO P 01.0408 L, METC 0105). In 2000, a random sample was drawn of patients registered at the Jan van Breemen Institute (now Reade) in Amsterdam, The Netherlands. Patients were eligible if they fulfilled the 1987 American College of Rheumatology (ACR) classification criteria, were diagnosed with RA between 1989 and 2001 and were aged 50 to 75 years. Patient enrollment was between 2001 and 2002 with follow up visits in 2004–2005 and 2010–2011. CV endpoints were defined as a verified medical history of coronary, cerebral or peripheral arterial disease (Table 1).

### DNA isolation and Taqman genotyping

Blood was obtained from 297 RA patients from the Radboudumc (RA1), 353 RA patients of the Jan van Breemen Institute (Reade)(RA2) and 234 individuals from the NBS NIMA study (NN). Genomic DNA was extracted from leukocytes in peripheral venous blood as previously described^55^. The samples were quantified and evaluated for purity (260/280-nm ratio) with a NanoDrop ND-1000 spectrophotometer (Thermo Scientific). The genetic variant in the *IL32* promoter (rs4786370) polymorphism was determined using the TaqMan SNP assay C_27972515_20, (Thermofisher, Foster City, CA, USA). The TaqMan qPCR assays were performed on the AB StepOnePlus polymerase chain reaction system (Applied Biosystems). Negative controls were included in the assay. No duplicates were used.

### IMT measurements

Carotid IMT was determined using an AU5 Ultrasound machine (Esaote Biomedica, Genova, Italy) with a 7.5 MHz linear-array transducer. Longitudinal images of the distal-most 10 mm of both the far wall and the near wall of both common carotid arteries (CCA) were obtained in the optimal projection. Actual measurement of the IMT was performed off-line by the sonographer at the time of the examination, using semi-automatic edge-detection software (M’Ath^®^Std Version 2.0, Metris, Argenteuil, France). Reproducibility of our IMT measurements as investigated by the method of Bland and Altman had been reported before: the mean ( ± S.E.M.) difference for repeated measurements by the same observer was 0.003 ± 0.007 mm 56,57.

### Statistical analysis

Normality was tested using the D’Agostino and Pearson omnibus normality test. Continuous variables are presented as mean and standard deviation (SD) or as median followed by the interquartile range (IQR). Categorical variables are presented as number followed by percentage. The differences between allele frequency and lipid concentrations measured in RA patients and individuals from the NN cohort were analyzed using the Mann-Whitney test. Chi-square test was used to test for differences between categorical variables. A *p-*value less than 0.05 was considered statistically significant (*p < 0.05 and **p < 0.01). Data was analyzed using GraphPad Prism v5.0.

## Additional Information

**How to cite this article**: Damen, M. S. M. A. *et al*. IL-32 promoter SNP rs4786370 predisposes to modified lipoprotein profiles in patients with rheumatoid arthritis. *Sci. Rep.* **7**, 41629; doi: 10.1038/srep41629 (2017).

**Publisher's note:** Springer Nature remains neutral with regard to jurisdictional claims in published maps and institutional affiliations.

## Supplementary Material

Supplementary Dataset 1

## Figures and Tables

**Table 1:** Baseline characteristics of the cohorts.

Cohort	NBS NIMA (NN)	Radboudumc (RA1)	Reade (RA2)
***N***	***234***	***297***	***353***
Age, years	61 ± 6	60 ± 12	63 ± 8
Female, no (%)	120 (51.34)	155 (52.2)	232 (65.7)
Disease duration, years	n.a.	9 (3–17)	7 (4–10)
Rheumatoid factor positive, no (%)	n.a.	202 (68.2)	256 (72.5)
Anti-citrullinated protein antibodies positive, no. (%)	n.a.	160 (53.9)	187 (54.5)
History of CVD, no (%)	57 (24.4)	63 (22.6)	51 (14.4)
DAS28	n.a.	2.97 (1.18)	3.90 (1.35)
Diabetes, no (%)	14 (6.0)	18 (6.1)	17 (4.8)
Systolic blood pressure, mmHg	130 ± 17	132 ± 17	142 ± 20
Diastolic blood pressure, mmHg	78 ± 10	77 ± 10	81 ± 9
Total cholesterol, mmol/L	5.92 ± 1.11	5.2 ± 1.13	5.77 ± 1.12
HDL cholesterol, mmol/L	1.39 ± 0.40	1.3 ± 0.34	1.46 ± 0.48
LDL cholesterol, mmol/L	3.89 ± 0.98	3.1 ± 1.07	3.69 ± 1.03
Triglycerides, mmol/L	1.29 (0.91–1.81)	1.53 (1.09–2.14)	1.32 (3.04–4.42)

Values are presented as mean ± SD, median (IQR) or numbers (percentages). CVD: cardiovascular disease, DAS28: disease activity score 28, HDL: high density lipoprotein; LDL: low-density lipoprotein.
