# Supplementary material for: IL-32 promoter SNP rs4786370 predisposes to modified lipoprotein profiles in patients with rheumatoid arthritis
Source: Sci Rep. 2017 Jan 30;7:41629. doi: 10.1038/srep41629 (PMC5278556; doi:10.1038/srep41629)
Supplement: Supplementary Dataset 1 [file srep41629-s1.doc]

**IL-32 promoter SNP rs4786370 predisposes to modified lipoprotein profiles in patients with rheumatoid arthritis**

*Michelle S.M.A. Damen^1^*, Rabia Agca^2^*, Suzanne Holewijn^3^, Jacqueline de Graaf^1^, Jéssica C. Dos Santos^1,4^, Piet L. van Riel^5^, Jaap Fransen^6^, Marieke J.H. Coenen^7^, Mike T. Nurmohamed^2^, Mihai G. Netea^1^, Charles A. Dinarello^1,8^, Leo A.B. Joosten^1^**, Bas Heinhuis^1^**, Calin D. Popa^5,6^**
*Authors share first authorship, **Authors share senior authorship
^1^Department of Internal Medicine and Radboud Center for Infectious Diseases (RCI), Radboud University Medical Center, 6525 GA Nijmegen, the Netherlands; ^6^Department of Rheumatology; ^7^ Radboud Institute for Health Sciences, Department of Human Genetics, Radboud university medical center, Nijmegen, The Netherlands; ^2^Amsterdam Rheumatology immunology Center, Department of Rheumatology, location CU University Medical Center and Reade, Amsterdam, The Netherlands; ^3^Rijnstate Ziekenhuis, Arnhem, The Netherlands; ^4^Instituto de Patologia Tropical e Saúde Pública, Universidade Federal de Goiás, Brazil; ^5^Department of Rheumatology, Bernhoven Ziekenhuis, Uden, The Netherlands; ^8^School of Medicine, Division of infectious diseases, University of Colorado Denver, Aurora, Colorado 80045, United States of America

Supplementary Fig. S1. DAS28-scores split on the IL-32 promoter SNP genotype within the two RA-patients cohorts. A. represents DAS28-scores of RA patients from the RA1 cohort. B. Represents DAS28-scores of RA patients in the RA2 cohort.
